# Computed tomography-based radiostereometric analysis achieves sufficient precision for femoral implants in total knee arthroplasty: a randomized controlled trial

**DOI:** 10.1186/s13018-025-06517-1

**Published:** 2025-11-28

**Authors:** Frank-David Øhrn, Lars H. W. Engseth, Are H. Pripp, Tommy Frøseth Aae, Anselm Schulz, Otto Schnell Husby, Stephan M. Röhrl

**Affiliations:** 1 Nordmøre and Romsdal Hospital, Møre and Romsdal Hospital Trust, Opdølvegen 4, 6450 Hjelset, Norway; 2 Faculty of Medicine and Health Sciences, Department of Neuromedicine and Movement Science (INB), NTNU Norwegian University of Science and Technology, Trondheim, Norway; 3 Division of Orthopaedic Surgery, Oslo University Hospital Ullevål, Oslo, Norway; 4 Faculty of Medicine, University of Oslo, Oslo, Norway; 5 Oslo Centre for Biostatistics and Epidemiology, Oslo University Hospital, Oslo, Norway; 6 Department of Radiology and Nuclear Medicine, Oslo University Hospital Ullevål, Oslo, Norway; 7 Faculty of Health Sciences, OsloMet – Oslo Metropolitan University, Oslo, Norway; 8 The Clinical Research Unit, Møre and Romsdal Hospital Trust, Ålesund, Norway

**Keywords:** CT-RSA, Total knee arthroplasty, Femoral implants, RSA, Radiostereometric analysis, Radiation dose

## Abstract

**Background:**

Conventional radiostereometric analysis (RSA) has been used for several decades to assess implant migration in total knee arthroplasty (TKA). More recently, computed tomography–based radiostereometric analysis (CT-RSA) has been introduced as an alternative. Experimental studies have demonstrated acceptable precision for CT-RSA; however, this has not yet been validated in a clinical setting. With the emergence of new alignment philosophies in TKA, migration analysis of femoral components has become increasingly important. A key criticism of CT-RSA has been the higher radiation doses compared with conventional RSA, particularly in longitudinal studies involving repeated CT scans. In this study, we evaluated the precision of CT-RSA for assessing femoral component migration in two TKA designs at two different radiation dose levels. We hypothesized that there would be no clinically relevant difference in precision between the two radiation dose levels.

**Methods:**

We performed a randomized controlled trial of 50 patients using Tritanium and GMK Sphere 3D Metal implants and analysed the precision of the method through double examinations expressed as means with 95% confidence intervals at standard and low-dose levels. The main outcome variable was the difference in mean maximum total point motion. We set the clinical meaningful difference to be more than 0.1 mm based on existing literature. Secondary outcome variables were the means and standard deviations of the translation and rotation of the implants.

**Findings:**

We found a precision of (mean, standard deviation) 0.20 (0.08) and 0.17 mm (0.06) for standard and low dose respectively, giving a difference (95% confidence interval) of − 0.03 mm (− 0.06–0.00). *P*-value was 0.031. Variability ratios were 0.009 for the standard deviation test.

**Interpretation:**

In this first clinical study to assess the precision of computed tomography–based radiostereometric analysis for femoral implants in total knee arthroplasty, we found no clinically relevant difference in precision. Our findings confirm that CT-RSA provides sufficient precision in a clinical setting to enable migration analysis of femoral implants in total knee arthroplasty, even when using very low radiation doses.

## Highlights

CT-RSA can be used to analyse migration of femoral implants in total knee arthroplasty.

## Background

Conventional radiostereometric analysis (RSA) has long been the gold standard for the migration analysis of orthopaedic implants [1–3], including those in the knee, hip and shoulder [4–6]. This method is noted for its high accuracy and precision.

However, conventional RSA involves considerable costs for calibration cages, tantalum beads, CAD models, and software licences. The insertion of tantalum beads during surgery is both time-consuming and invasive, and the subsequent image analysis is technically demanding, requiring specialized training. In recent years, computed tomography-based RSA (CT-RSA) has emerged as a promising alternative. Several studies have shown that CT-RSA provides high accuracy and precision for hip and shoulder implants [7–9]. Our research group has published a series of experimental studies demonstrating that CT-RSA of knee implants can achieve comparable, if not superior, precision to conventional RSA [10–12]. However, a key criticism of CT-RSA has been the higher radiation doses compared with conventional RSA, particularly in longitudinal studies involving repeated CT scans. [13]. Nevertheless, CT scans are more readily available globally and quicker to perform [11, 12]. Additionally, analysing CT-RSA images is more efficient than analysing conventional RSA images [12]. Conventional RSA can be used to assess migration of femoral components in total knee arthroplasty (TKA), but this is technically challenging. The geometry of femoral implants makes it difficult to insert tantalum beads in the distal femur without the markers being obscured by the implant. This challenge is further amplified by the increasing adoption of new alignment philosophies in TKA, such as kinematic and functional alignment, which adjust the femur in the coronal and axial planes to achieve a more personalized fit. These approaches may affect implant fixation and increase the relevance of precise migration analysis of the femoral component.

In a previous cadaver study, we demonstrated that CT-RSA of femoral components in TKA is both feasible and precise, even at low radiation doses [14]. The aim of the present study was to evaluate the precision of CT-RSA in a clinical setting. We hypothesized that the precision would be sufficient for clinical use.

## Methods

The present study was part of the “Comparison of the in vivo stability of 2 cementless TKA designs using CT micromotion analysis—A randomized controlled trial” (Cless*TKA*) study and thus used the same sample size. 50 patients between the ages of 50 and 75, with knee osteoarthritis (Kellgren and Lawrence grade 3 or 4) and requiring knee replacement surgery, were randomized to receive either a GMK Sphere 3D Metal (Medacta International, Switzerland) or a Triathlon Tritanium (Stryker, Mahwah, USA) TKA. Exclusion criteria were rheumatoid arthritis or other inflammatory joint diseases, body mass index (BMI) > 35 kg/m^2^, > 15 degrees of varus or 5 degrees of valgus on standing long-leg x-rays, ongoing cancer treatment, serious psychiatric disorders, inability to speak or read Norwegian or the use of walking aids for reasons other than knee osteoarthritis. The randomization was performed using the EFORSK software (Unit for Applied Clinical Research, NTNU, Norway) with varying block sizes and stratified by sex. No more than one week prior to surgery, one of the authors (FDØ) performed the randomization. The patients, study nurse and physiotherapists were all blinded to the type of implant. The randomization codes were used in both the surgery planning programme (A or B) and the patient file, thereby ensuring blinding. The patients and the study nurse had to confirm at each control that they still were blinded. The statistician was also blinded to implant type and dosage level. All surgeries were performed at Kristiansund Hospital from January to June 2023 by the same two surgeons (FDØ/OSH), who consistently worked as a team. All postoperative controls were performed at the same location. Patella resurfacing and mechanical alignment were performed in all cases without using a tourniquet. All complications were accounted for. Double CT acquisitions were performed postoperatively within 1–2 days of surgery for the standard dose with an effective dose (ED) of 0.05 mSV per scan, and at 12 months postoperatively for the low dose with an ED of 0.01 mSv per scan. The EDs were calculated using the knee conversion factor of 0.0004 [11, 15]. CT scans were conducted using the same GE Revolution CT scanner (GE Healthcare, Chicago, USA) at all time points. Analyses were performed using CTMA Software (Sectra, Linköping, Sweden) with 150 HU (bone, low smoothing) and 3000 HU (metal, no smoothing), and a bone + metal artefact reduction (MAR) reconstruction algorithm (Table 1). Both the femoral bone and the metal implants were selectively marked (Figs. 2 and 3). Axes were set as shown in Fig. 4. Peripheral points labelled as anterior, medial, lateral, posterolateral and posteromedial were created in the software (Fig. 4). Demographic data such as BMI, sex and age were collected. The primary outcome measure, the mean of the maximum total point motion (MTPM), representing the point on the implant surface that moved the most between the two examinations, was calculated, along with the secondary outcome measures, mean of the centre of mass (COM) and translations and rotations of peripheral points. Translations and rotations in all planes were given the clinically meaningful names transversal, posterior and proximal translations and transversal, varus and internal rotations. This was done to avoid confusion as there is a difference in the orientation of the coordinate systems of CT-RSA and conventional RSA [13, 16]. Double examinations were performed a few minutes apart, with the patient supine and repositioned in the reset CT scanner. All the CT-RSA analyses were performed by one of the authors (FDØ), who was certified by Sectra and has performed analyses in previous studies [10, 11, 14].

**Table 1:** CT protocol and CTMA protocol of the study

CT scans	Standard dose	Low dose
CT scanner	GE revolution	GE revolution
Tube voltage (kV)	120	120
Rotation time	0.5 s	0.5 s
Pitch	1.0	1.0
Scan length (mm)	180	180
Tube current (mAs)	160	40
Effective dose (mSv)	0.05	0.01
*CT-RSA analysis*		
CT-RSA software	CTMA	CTMA
Reconstruction algorithm	Bone + MAR	Bone + MAR
Bone smoothing	Low	Low
Metal smoothing	None	None
Bone HU value	150	150
Implant HU value	3000	3000

**Fig. 1:**
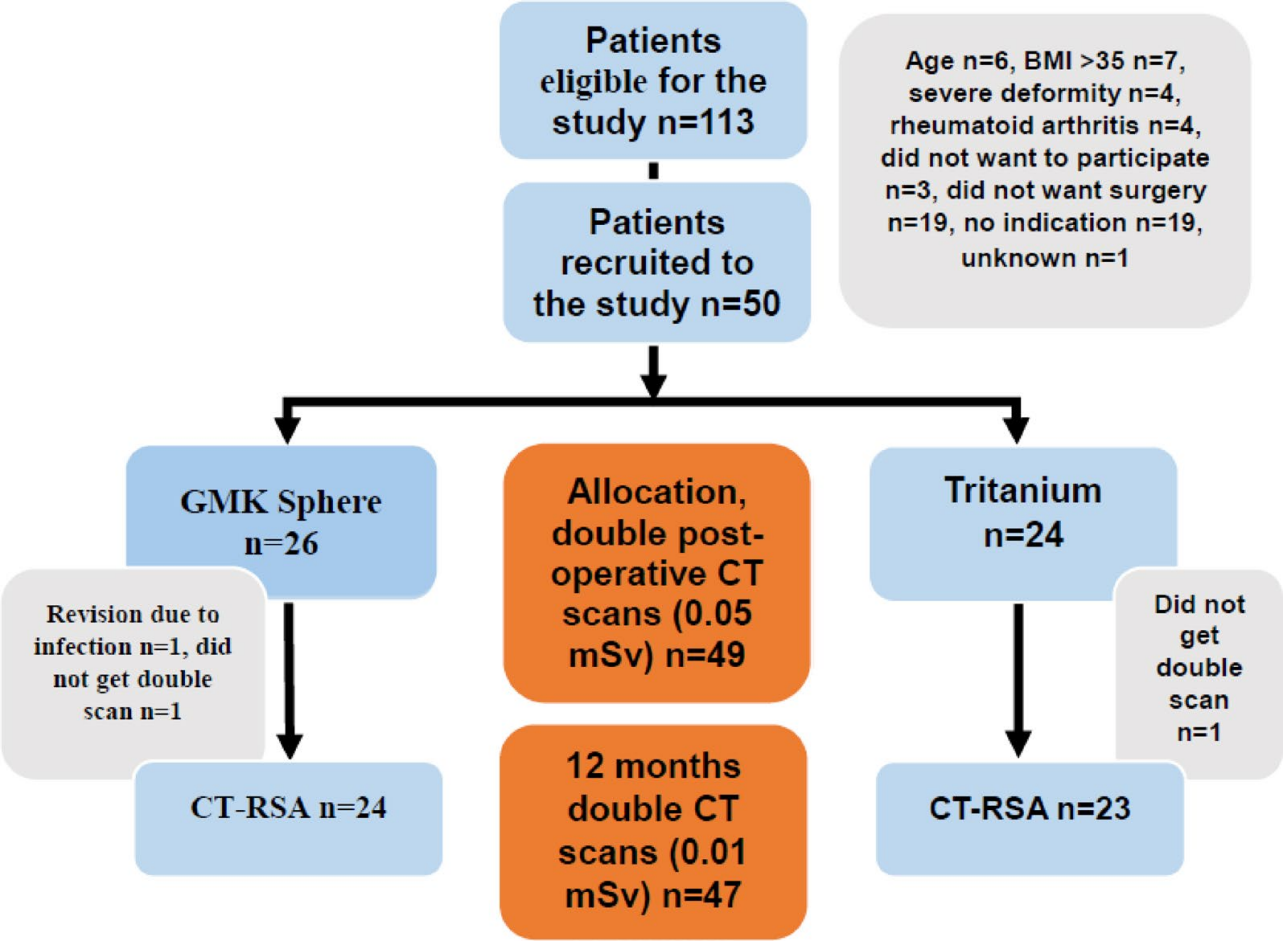
Flowchart of the study

**Fig. 2:**
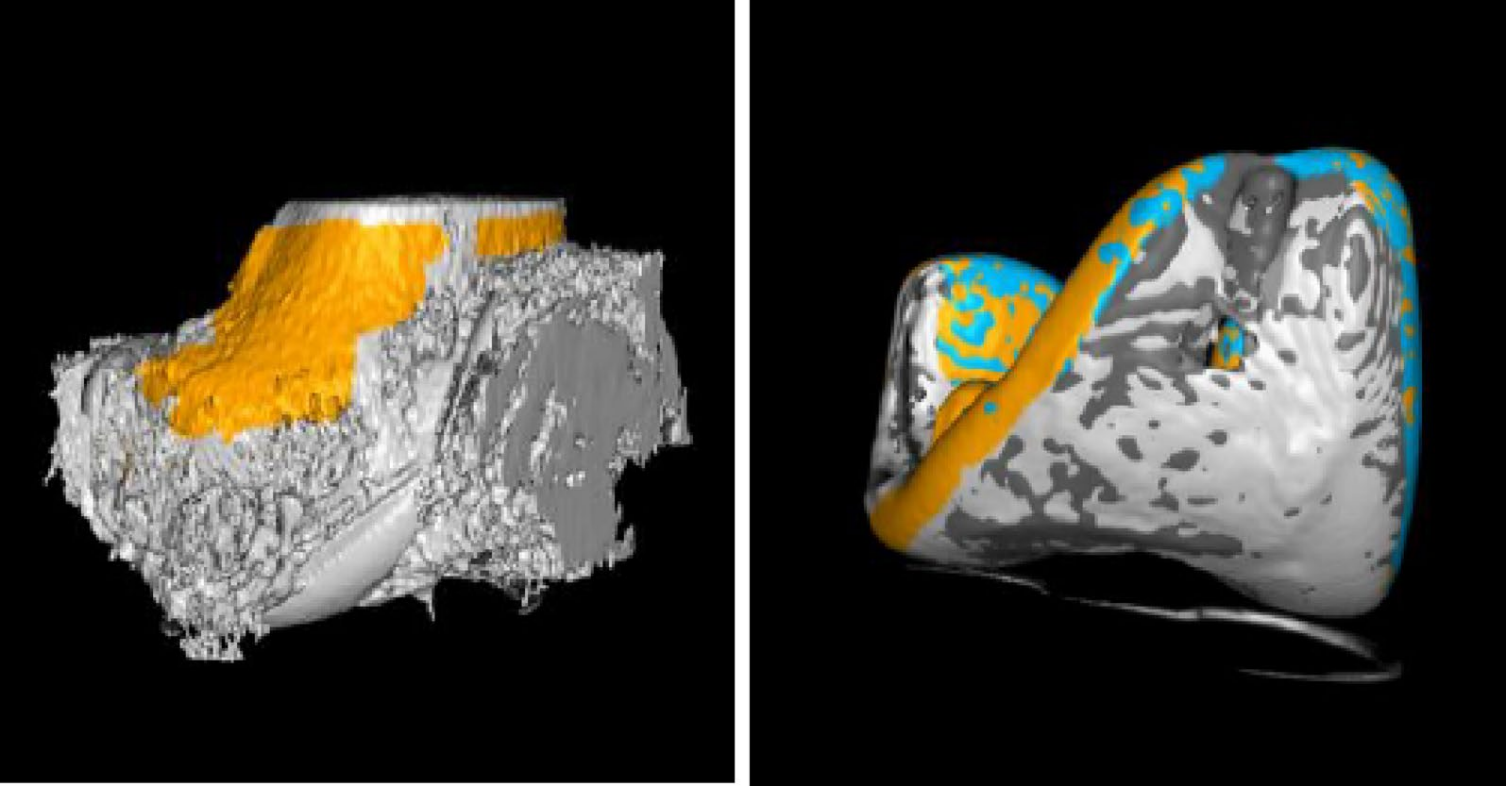
Selective marking of the femoral component and femoral bone

**Fig. 3:**
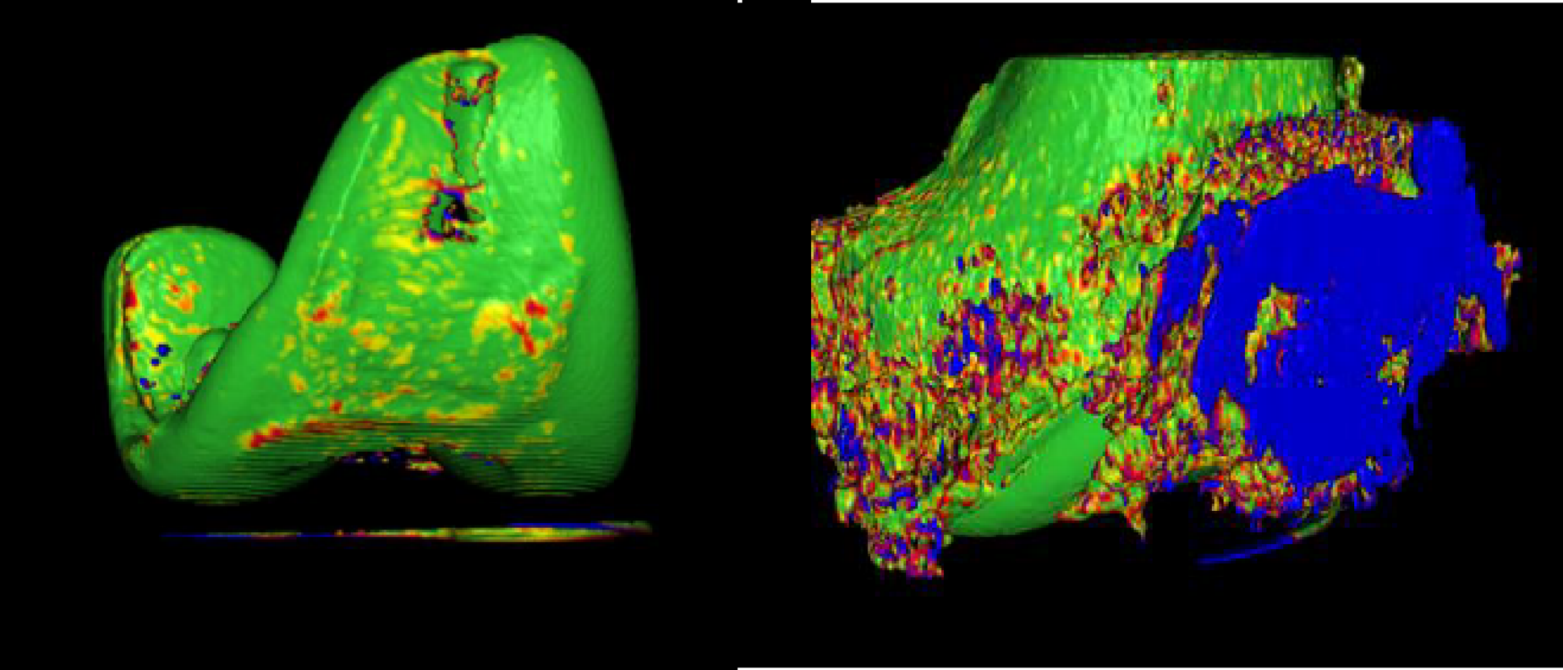
Digital CTMA models of the femoral implant and femoral bone (HU 3000 vs. 150)

### Statistics

All statistical analyses were conducted using STATA version 17 (StataCorp LLC, College Station, TX, USA). Means, standard deviations (SD), and 95% confidence intervals (CI) were calculated for the variables. We calculated and compared the means of continuous demographic using the independent samples t test. Linear mixed model analyses of contrasts between MTPM, COM and translations and rotations of peripheral points were performed. The analysis included a fixed factor (standard and low dose) and patients as random effects. The means of the calculations from the double examinations represented the bias, and the SD represented the random error [16]. A migration of more than 0.2 mm per year is known as an important threshold for implant loosening. In our previous cadaver studies we thus stated that a mean difference of precision (MPTM) of > 0.1 mm was considered clinically relevant [1, 11, 14]. Thus, if there was a difference of < 0.1 mm, it would not be considered to be clinically relevant even if *p* < 0.05. Variability ratios were calculated using the SD test command in STATA.

## Results

**Fig. 4:**
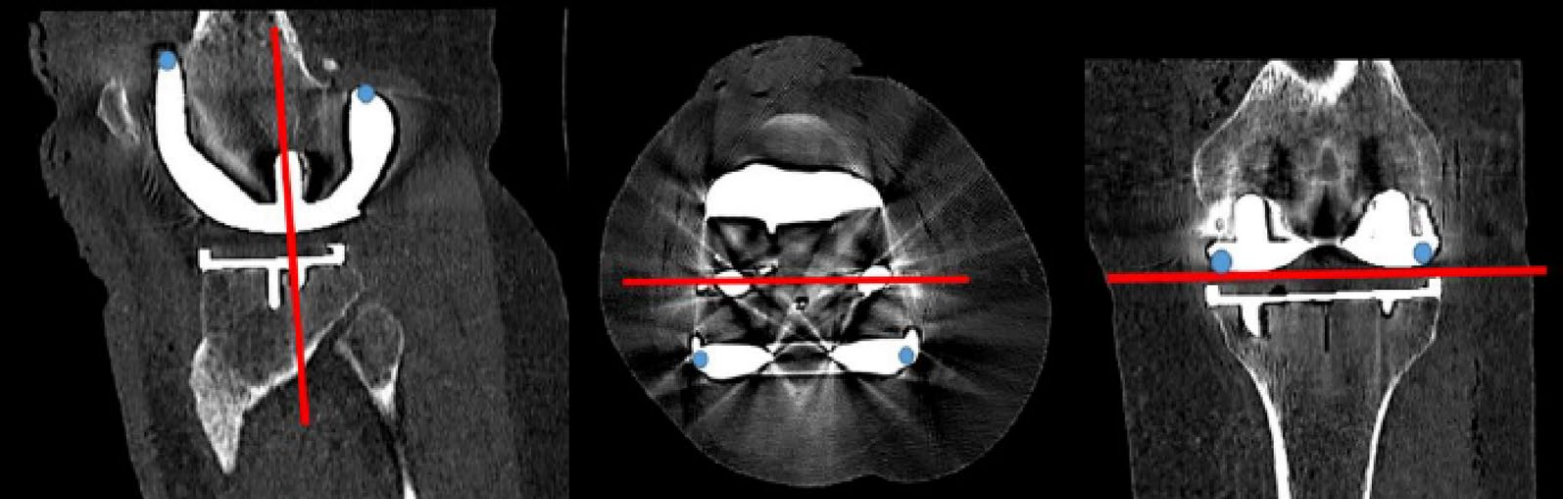
Multiplanar reconstruction axes set in the CTMA software and the peripheral points named anterior, medial, lateral, posterolateral and posteromedial

**Fig. 5:**
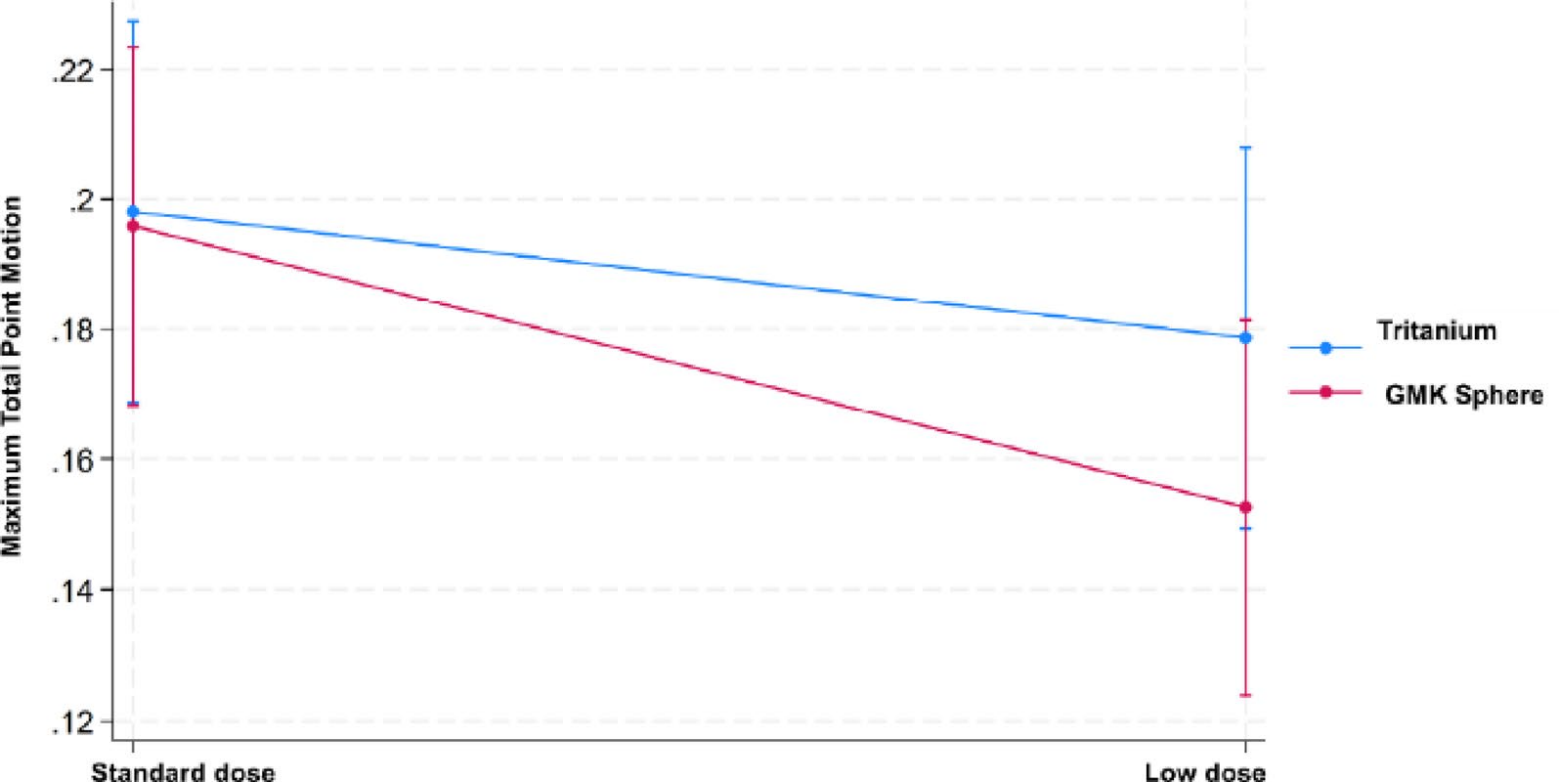
Comparison between the mean MTPM with 95% CI of standard and low dose

Demographic data are reported in Table 2. The CT-RSA analysis showed a mean difference between standard (SD = 0.08) and low (SD = 0.06) dose of 0.03 mm MTPM (95% CI − 0.06 to 0.00, *p* = 0.031) for all implants, with an SD test of *p* = 0.009. The mean difference in MTPM between the Tritanium (SD = 0.07) and GMK Sphere (SD = 0.08) was − 0.01 mm (95% CI − 0.04 to 0.02, *p* = 0.346), with an SD test of *p* = 0.893. For further details, please see Tables 3 and 4 and Figs. 4 and 5.

**Table 2:** Demographics

Variables (n = 50)	Tritanium n = 24	GMK Sphere n = 26	*P* value
Age (years, mean, 95% CI)	64.2 (61.1–67.3)	66.8 (64.5–69.2)	0.129
BMI (kg/m^2^, 95% CI)	29.4 (27.8–31.0)	28.3 (26.8–29.9)	0.688
HKA (°, 95% CI)	175.4 (172.7–178.1)	176.1 (174.0–178.2)	0.417
Male/female (n)	10/14	11/15	
Left/right (n)	12/12	11/15	
Implant (n)	24	26	

**Table 3:** Mean translations and rotations of the femoral component when using standard and low dose

	Standard dose, mean n = 48 (SD)	Low dose, mean n = 47 (SD)	Difference (95% CI)	*P* value	SD test
MTPM	0.20 (0.08)	0.17 (0.06)	− 0.03 (− 0.06 to 0.00)	0.031	0.009
*COM translations (mm)*					
TT	0.08 (0.08)	0.07 (0.03)	− 0.02 (− 0.03 to 0.00)	0.032	0.034
Transversal	0.00 (0.04)	0.00 (0.02)	0.01 (− 0.01 to 0.02)	0.376	0.000
Posterior	0.00 (0.06)	− 0.01 (0.05)	− 0.01 (− 0.03 to 0.01)	0.394	0.126
Proximal	0.00 (0.06)	0.01 (0.05)	0.00 (− 0.02 to 0.02)	0.872	0.205
*Rotations (º)*					
TR	0.19 (0.10)	0.16 (0.08)	− 0.03 (− 0.06 to 0.00)	0.08	0.009
Transversal	0.01 (0.16)	− 0.01 (0.10)	− 0.02 (− 0.07 to 0.03)	0.439	0.002
Varus	− 0.01 (0.11)	− 0.01 (0.11)	− 0.01 (− 0.05 to 0.04)	0.796	1.068
Internal	− 0.02 (0.10)	0.02 (0.09)	0.04 (0.00 to 0.07)	0.032	0.777
*TT peripheral points*					
Anterior	0.12 (0.07)	0.10 (0.05)	− 0.02 (− 0.04 to 0.00)	0.102	0.021
Medial	0.16 (0.08)	0.12 (0.05)	− 0.04 (− 0.06 to− 0.01)	0.009	0.002
Lateral	0.14 (0.07)	0.12 (0.05)	− 0.02 (− 0.04 to 0.01)	0.102	0.048
Posterolateral	0.11 (0.06)	0.10 (0.06)	− 0.01 (− 0.04 to 0.01)	0.223	0.851
Posteromedial	0.11 (0.08)	0.10 (0.05)	− 0.02 (− 0.04 to 0.01)	0.180	0.011

SD, standard deviation; TT, total translation; TR, total rotation; CI, confidence interval; n, number of patients

**Table 4:** Translations and rotations of the femoral component when using Tritanium versus GMK Sphere (standard dose)

	Tritanium, mean n = 23 (SD)	GMK Sphere, mean n = 26 (SD)	Difference (95% CI)	*P* value	SD test
MTPM	0.19 (0.07)	0.18 (0.08)	− 0.01 (− 0.04 to 0.02)	0.346	0.893
*COM translations (mm)*					
TT	0.08 (0.04)	0.07 (0.04)	− 0.01 (− 0.02 to 0.01)	0.300	0.439
Transversal	0.00 (0.04)	0.00 (0.03)	0.00 (− 0.01 to 0.01)	0.992	0.098
Posterior	− 0.01 (0.06)	0.00 (0.05)	0.01 (− 0.01 to 0.04)	0.261	0.514
Proximal	0.01(0.06)	0.00 (0.06)	− 0.01 (− 0.03 to 0.02)	0.568	0.644
*Rotations (º)*					
TR	0.18 (0.11)	0.18 (0.08)	− 0.01 (− 0.04 to 0.03)	0.762	0.184
Transversal	− 0.02 (0.12)	0.01 (0.14)	0.03 (− 0.02 to 0.09)	0.266	0.217
Varus	− 0.01 (0.11)	− 0.01 (0.11)	0.00 (− 0.05 to 0.04)	0.899	0.875
Internal	0.00(0.12)	0.00(0.13)	0.00 (− 0.04 to 0.04)	0.963	0.037

### Complications

One patient in the study group was revised and thus excluded due to a postoperative deep infection, one patient in the control group went through soft tissue debridement and irrigation procedure due to a postoperative hematoma. Furthermore, one patient in the study group and two patients in the control group suffered from postoperative stiffness and went through mobilisation under anaesthesia. A few patients from each group had minor wound problems or palpable stay sutures from the knee capsule removed. Their problems all resolved.

### Missing data

**Fig. 6:**
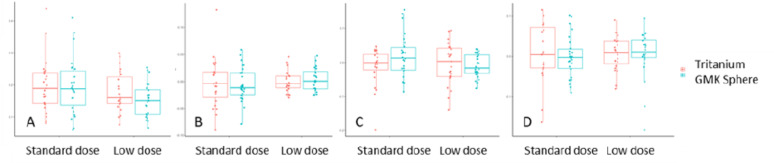
Boxplots of the MTPM^A^, transversal^B^, posterior^C^ and proximal^D^ translations

The double examinations with the standard dose were performed postoperatively, while the double examinations with the low dose were performed at 12 months. Consequently, the patient who developed an infection and was excluded has missing data for the low-dose group. In addition, one patient is missing double examinations postoperatively, and two patients are missing them at the 12-month follow-up. Further details are shown in Fig. 6.

## Discussion

Our main finding was that precision levels were equal or superior to those found in existing literature on conventional RSA. There was also equal precision between the two dosage levels. Although some of the *P*-value s of the study showed a significant difference in precision in favour of the low dose CT-RSA, the actual differences were smaller than the margin of clinical relevance of 0.1 mm and are thus not relevant in clinical practice. The MTPMs found in the present study were slightly lower than in the previously published porcine cadaver study (0.20 and 0.17 mm vs. 0.25 vs. 0.29 mm), despite the fact that the low radiation dose was even lower (0.01 mSv vs. 0.02 mSv) in the clinical study [14]. Variability was on approximately the same level (0.08 and 0.06 vs. 0.10 and 0.08), although we had 50 unique, independent samples in this clinical study. We found that the typical mean precisions were very low for the translations and rotations in COM, with values close to 0. The MTPM, total translation (TT) and total rotation (TR) values were slightly higher and typically ranged between 0.1 and 0.2 mm or degrees for most of the values. However, this is not surprising, as these vectors cover movement in three directions, unlike the other translations and rotations that only cover one dimension at a time. Our results are also comparable to the precision of conventional RSA of TKA in the literature. Christensson et al. reported mean (SD) MTPM values of 0.22 mm (0.14) for the tibial and 0.15 mm (0.10) for the femoral components [17]. Koster et al. found higher values for Persona components: tibial 0.49 mm (0.26) and femoral 0.34 mm (0.18), compared with NexGen LPS: tibial 0.32 mm (0.21) and femoral 0.24 mm (0.12) [18]. Furthermore, for Attune and LCS components, mean (SD) MTPM values ranged from 0.24 mm (0.20) to 0.30 mm (0.14) [19]. Yüksel et al. reported even higher values for Attune and PFC Sigma components, with means (SD) between 0.41 mm (0.23) and 0.71 mm (0.62) [20]. Not all authors report precision of femoral components derived from double examinations. Niesen et al. found tibial component values of 0.18 mm (0.11) and 0.21 mm (0.13) [21]. Overall, tibial component precision typically ranged from approximately 0.2–0.5 mm, and femoral component precision from 0.15 to 0.35 mm, with some higher outliers. Our results are therefore on level with, or better than, previously published precision data for conventional RSA. In addition, none of these studies reported precision in terms of TT of COM, TR, or TT of peripheral points.

Migration of femoral components is worthy of greater attention now that alternative alignment philosophies have gained popularity, such as kinematic alignment, inverse kinematic alignment and functional alignment [22–25]. In these new surgical techniques, the tendency is to align the femoral component more in varus or valgus and more internally rotated compared to the mechanical axis of the limb. Mechanical alignment has been the gold standard for TKA surgical techniques for many decades, as the aim was to ensure equal load transfer over the entire implant and therefore reduce aseptic loosening. The new alignment philosophies challenge these established tenets of load transfer across the implant in unforeseen and under-researched ways. These changes in load transfer may affect the migration of femoral implants, which warrants precise methods of measuring their micromotion.

In the present publication, we used EDs of 0.05 and 0.01 mSv. These were even lower than in our previous cadaver study, where we used EDs of 0.08 and 0.02 mSv respectively [14]. Although the radiation dose was lower, our results on precision were still superior. The low dose radiation in the low dose CT-RSA protocol used, was lower than in a previous clinical study using conventional RSA of tibial implants. In that study we documented an ED of 0.016 mSv for RSA [12]. In conventional RSA studies, one often has to repeat image acquisition to obtain adequate images, especially for femoral components to avoid hidden markers. Hence conventional RSA probably yields higher total EDs. Retakes are seldomly necessary with CT-RSA. One of the reasons for the minimal radiation exposure in CT-RSA is the exceptionally low conversion factor of 0.0004 [15]. This is attributed to the distance of the knee from the body’s radiation-sensitive organs. Nevertheless, the findings of this study are also relevant to other joints. Another reason why we can use such low doses is that the CTMA software uses surface models of the bone and implants. We do not need to visualize the soft tissue or the cancellous bone. Minimizing radiation exposure is especially critical, as patient participation in this study was for research purposes only, without any direct clinical benefit for the patients involved. In addition, migration studies typically include 5–7 scans during a two-year study period. These scans contribute to the total lifetime radiation exposure from medical imaging, which can be substantial and thus increase the risk of cancer. Therefore, any radiation exposure should be as low as reasonably possible [26]. Our publication shows that low radiation doses do not compromise the precision of the CT-RSA method for femoral implants.

### Weaknesses

Our study had a few limitations. First, the CT scans were performed using a low-radiation protocol, which meant that they were kept as short as possible and terminated just above the femoral implant. If the scans had included a longer segment of the femur, the precision results might have been even more robust.

Second, although the patients, study nurse, physiotherapist, and statistician were blinded to the treatment, the surgeons (FDØ/OSH) were of course not blinded. Similarly, the person conducting the analyses (FDØ) was not blinded. We acknowledge this as a weakness to the study and a potential source of bias, however, when the study commenced, FDØ was one of very few people internationally that mastered the analysis of CT-RSA. We do however not think this affected the results and conclusion of the study. Furthermore, the study is a single centre study, and this might of course affect the generalizability of the results. Nevertheless, our inclusion criteria are broad and the exclusion criteria of the study are few, hence the external validity should be high. Also, we did not perform a sample size calculation on this study, as it is part the Cless*TKA* study. However, according to the recommendations of migration studies recently published, a minimum of 25% of the patients in a migrations study should perform double examinations [16]. This would typically include at least 15 patients. In the present study, all 50 patients underwent double examinations at postoperative. Three patients were missing a double low-dose examination at 12 months: one had revision surgery, and two missed the second examination due to error. The impact of these missing data on the results is unknown. However, compared with conventional RSA studies, where substantially more cases are often lost due to hidden markers, high mean error, or high condition numbers, we think the acquisition of 47 out of 50 patients at 12 months represents an excellent outcome. [25, 27].

### Strengths

To our knowledge, this is the first study to evaluate the precision of CT-RSA of femoral components in TKA in a clinical setting. This is important, as our previous investigation was conducted on a porcine cadaver and involved repeated analyses of a single sample only, which does not constitute independent sampling [14]. Experimental or phantom studies are generally used for evaluation under optimized conditions. The current study, being a randomized controlled trial with similar demographics in both groups (Table 2) confirms the conclusions drawn from previous cadaver-based research. Furthermore, we were able to evaluate precision in two different implant designs. Given that implant geometry and metal alloy composition can vary, there is a theoretical possibility of differing artefact levels and, consequently, variability in precision [28]. This study was conducted in accordance with established migration analysis guidelines [16]. We therefore calculated the mean differences in MTPM and across all six degrees of freedom for various dosage levels and implant types. Standard deviations were also reported as indicators of random error.

## Conclusion

In this first clinical study to assess the precision of CT-RSA for femoral implants in total knee arthroplasty, we found no clinically relevant difference in precision. Our findings confirm that CT-RSA provides sufficient precision in a clinical setting to enable migration analysis of femoral implants in total knee arthroplasty, even when using very low radiation doses.

## Data Availability

The datasets generated and/or analysed during the current study are not publicly available but are available from the corresponding author on reasonable request.
